# PD-1 and beyond to Activate T Cells in Cutaneous Squamous Cell Cancers: The Case for 4-1BB and VISTA Antibodies in Combination Therapy

**DOI:** 10.3390/cancers13133310

**Published:** 2021-07-01

**Authors:** Quentin Wright, Jazmina L. Gonzalez Cruz, James W. Wells, Graham R. Leggatt

**Affiliations:** The University of Queensland Diamantina Institute, The University of Queensland, Woolloongabba, QLD 4102, Australia; q.wright@uq.edu.au (Q.W.); j.gonzalezcruz@uq.edu.au (J.L.G.C.); j.wells3@uq.edu.au (J.W.W.)

**Keywords:** skin cancer, squamous cell carcinoma, VISTA, PD-1, 4-1BB

## Abstract

**Simple Summary:**

The use of checkpoint antibodies has revolutionized the treatment of cancer. Tumor-infiltrating T cells, key mediators of anti-tumor immune responses, are often actively silenced by the tumor microenvironment. Checkpoint antibodies block inhibitory signals or enhance positive signaling pathways in these T cells to overcome silencing, resulting in an improved anti-tumor T-cell response. To date, many clinical studies have focused on blocking inhibitory pathways (e.g., CTLA-4 and PD-1), with varying success. Increasingly, alternative checkpoint molecules are being identified and used as monotherapies, or in combination with existing PD-1/CTLA-4 treatments. This review dissects the potential role of checkpoint antibodies against PD-1, VISTA and 4-1BB in the future treatment of cutaneous skin cancers.

**Abstract:**

Non-melanoma skin cancers (NMSC) have a higher incidence than all other cancers combined with cutaneous squamous cell carcinoma (cSCC), capable of metastasis, representing approximately 20% of NMSCs. Given the accessibility of the skin, surgery is frequently employed to treat localized disease, although certain localities, the delineation of clear margins, frequency and recurrence of tumors can make these cancers inoperable in a subset of patients. Other treatment modalities, including cryotherapy, are commonly used for individual lesions, with varying success. Immunotherapy, particularly with checkpoint antibodies, is increasingly a promising therapeutic approach in many cancers, offering the potential advantage of immune memory for protection against lesion recurrence. This review addresses a role for PD-1, 4-1BB and VISTA checkpoint antibodies as monotherapies, or in combination as a therapeutic treatment for both early and late-stage cSCC.

## 1. Introduction

Keratinocyte cancers, consisting of squamous cell carcinoma (SCC) and basal cell carcinoma (BCC) are the most frequently diagnosed cancers in fair-skinned populations. Recent studies revealed that approximately 5.4 million keratinocyte cancers were diagnosed and treated in 3.3 million patients in the USA in 2012, with mortality rates increasing in recent years (2011–2017) [1,2]. In Australia, the person-based incidence rates of keratinocyte cancer excisions (BCC and SCC collectively) were 1531 per 100,000 person-years, of which Queensland had the highest recorded incidences [3]. These cancers are primarily driven by cumulative exposure to the ultraviolet component of sunlight, which generates a high mutational burden within keratinocyte DNA [4]. The most common mutations are in the *p53*, *patched*, and *ras* genes, all of which regulate the cell cycle. While basal cell carcinomas are more frequent than squamous cell carcinoma, it is SCC that is more likely to result in aggressive disease that metastasizes from the skin, and will therefore form the focus of this review.

Actinic keratosis (AK), a hyperproliferative epithelial lesion, is believed to be a precursor to the development of cutaneous SCC, with variable estimates from 0.025% to 16% for progression of an individual AK to SCC per year [5,6,7]. Multiple AKs can often occur simultaneously in the same patient, with higher progression rates often associated with patients with multiple lesions. Actinic keratosis typically presents as abnormally proliferative keratinocytes restricted to the epidermis, while progression to SCC involves disruption of the basement membrane and penetration of tumor cells into the underlying dermis and beyond. The current treatment of AK or cutaneous SCC (cSCC) can involve surgical excision or cryosurgery for individual lesions, or laser techniques and topical creams, such as imiquimod or 5-Fluorouracil, for the treatment of an entire field [8].

While surgical excision is frequently curative for localized SCC, patients can have multiple simultaneous lesions in regions such as the face, unresectable tumors, recurrent tumors, or invasive SCC that spreads to other body organs, leading to fatal disease [9]. Therapeutic alternatives to surgery that result in long-lasting protection across the entire skin surface would be ideal. In this regard, harnessing the innate and adaptive immune response to attack cSCC from the inside, through immunotherapeutic approaches, has the potential to establish not only effector, but also memory, responses, thus reinstating tumor-specific immunosurveillance.

## 2. Natural Immunity to cSCC

Evidence for a natural immune surveillance of cSCC comes from studying immunosuppressed individuals, who take medication on a daily basis to prevent organ rejection and have a greatly enhanced risk of skin SCC development [10]. Organ transplant patients have a 65–250-fold increased risk of cutaneous SCC incidence, with the variation likely due to the nature, intensity and duration of the immunosuppressive drug that is administered [11]. In immunocompetent individuals, tumor-infiltrating lymphocytes and other inflammatory cells are often observed within cSCC [12,13,14,15,16]. This includes CD8 T cells that are specific for UV-mutated proteins, such as p53 [13]. Using single-cell RNA-seq and spatial transcriptomics, one comprehensive study characterized inflammatory cell populations, including macrophages, myeloid-derived suppressor cells, and exhausted T cells, at the leading edge of the tumor mass [15]. Even at the precancerous stage of AK, disruption to the keratinocyte cell cycle and UV-induced oxidative stress can induce the production of chemokines and cytokines, leading to leukocyte recruitment and a chronic inflammatory environment. For example, CCL27 and CCR10, a chemokine axis involved in lymphocyte migration to the skin, was found to be upregulated in actinic keratosis and cSCC [17]. One study found that the inflammatory cytokine IL-6 was upregulated in AK compared with uninvolved skin [18]. In addition, UV-induced damage is known to activate inflammasome activity, resulting in the release of active IL-1 family members to enhance inflammation [19,20]. In contrast, several studies have shown a downregulation of the inflammasome proteins ASC and NLRP1 within cSCC, suggesting that inflammasome activity might decrease during the progression from AK to SCC [21,22]. The presence of an immune cell infiltrate in AK and cSCC, without clearance of the tumor, suggests that the tumor microenvironment may be suppressive. Certainly, in sun-exposed regions of the skin, UV light has been shown to be generally immunosuppressive to new immune responses within the skin, making it more difficult to dissect the suppressive contribution of the developing tumor without direct comparison with neighboring, photodamaged skin [23]. The expression of proteins involved in the type 1 interferon signaling pathway have been shown to be downregulated in AK [24]. Increasing numbers of CD4^+^FoxP3^+^ T cells (Treg) have been demonstrated during SCC development, along with suppressive cytokines such as IL-10 and TGF-β [25,26,27]. However, another study examining photodamaged skin, intraepithelial carcinoma (IEC), and SCC by flow cytometry did not show an enrichment of Treg with tumor stage [28]. While the discrepancy in Treg enrichment might relate to different patient cohorts or methods used for analysis, the functional activity of Tregs at different stages of SCC will also be an important parameter for future studies. While there are clearly multiple cellular and soluble pathways to immune suppression in cSCC, consideration must also be given to ligand/receptor interactions on the surface of both tumor cells and effector T cells, which can alter the behavior of anti-tumor T cells. On T cells, these ligands can be subdivided into inhibitory and activating receptors, with PD-1 and CD28, respectively, being key examples of each [29]. The modulation of these receptors, through agonist or blocking antibodies, has become a cornerstone of modern cancer immunotherapy and is referred to as immune checkpoint therapy [30]. Within cutaneous SCC, the expression of inhibitory and activating receptors at different stages of disease progression has been understudied in humans, with perhaps the exception of PD-1 and its ligand PD-L1. Given that immune checkpoint antibodies often work best on cancers with high mutational load, and therefore plenty of potential neoantigens for T-cell receptor (TCR) recognition, understanding the expression of these antibody targets in cutaneous SCC might lead to new therapeutic avenues [4].

## 3. Immunotherapy in cSCC

Immunotherapy is an emergent field of medicine, based on boosting the strength of the immune system to effectively treat cancer. In keratinocyte cancers, the most prominent immunotherapy has been topical imiquimod, which engages TLR7 within the skin to invoke a strong, local inflammatory response [31]. This inflammatory response alone has variable success in clearing primary SCC, and the induction of T-cell responses has rarely been analyzed. With regard to T-cell responses, there are several promising treatment modalities, including adoptive cell therapies (CAR T cells) [32], vaccines [33] and checkpoint therapies [34]. Strategies involving adoptive cell therapy or vaccines generally require the identification of target tumor antigens. Mutations within human cSCC are frequent, but the identification of tumor-specific peptides that bind to MHC or surface-exposed tumor antigens in cSCC needs further study. In contrast to cellular therapies or vaccines, the administration of checkpoint antibodies does not require prior knowledge of the tumor antigen. Tumor-infiltrating lymphocytes are primed to available tumor antigens, and the blocking of inhibitory co-receptors or the stimulation of activating co-receptors enhances their response. Proof of principle for this approach in cSCC has come from human clinical trials of anti-PD-1 antibody therapy in metastatic or locally advanced cSCC, where around 40% of patients showed regression of tumors [35]. Widespread use of checkpoint antibodies in cSCC, particularly early cSCC, has been limited by the effectiveness of surgery in primary tumors as a cure, the high costs associated with checkpoint antibodies, and the unwanted systemic toxicities associated with some antibodies. However, not all AK or primary cSCC are readily removed by surgery and tumor recurrence can be problematic, suggesting that alternatives are needed as primary or supportive therapies. The high costs and systemic toxicities of these treatments may be circumvented by approaches that localize the antibodies to the tumor [36]. Consequently, the remainder of the review will look at promising checkpoint candidates to be considered in cSCC, and speculate on the effective delivery of checkpoint antibodies to epithelial tumors.

## 4. Programmed Death- 1 (PD-1)

Programmed death protein 1 (PD-1) signaling is a negative regulator of T-cell function, most commonly associated with T cells exposed to chronic/repetitive antigen [37,38,39]. A role for PD-1 signaling was identified in chronic LCMV infection, where the function of exhausted T cells could be recovered by blocking PD-1 interaction with PD-L1 [40,41]. Similar observations were also evident in dysregulated T cells during anti-tumor immune responses [42]. This checkpoint mechanism likely evolved to limit autoimmunity and autoreactive T cells, as PD-1 knockout mice can develop spontaneous autoimmunity and PD-1 deficiency accelerates disease in autoimmune-prone mice [39,43]. PD-1 is expressed on CD4 and CD8 T cells, along with several other cell types, including NK cells, B cells and macrophages, and typically is upregulated on T cells upon activation [44,45]. After the binding of PD-1 to its ligand PD-L1/L2, expressed on hematopoietic cells and cancer cells, the suppression of T-cell responses is facilitated by the recruitment of the SHP-1/SHP-2 phosphatases, leading to reduced TCR and CD28 intracellular signaling, in addition to the downregulation of down-stream transcription factors and T-cell-secreted cytokines [37,39]. The reduction in effector T-cell response, mediated by PD-L1/L2, can be moderated by the presence of alternative, competing ligands, such as B7(CD80) and RGMB for PD-L1 and PD-L2, respectively [46]. In addition, a recent study has suggested that the engagement of PD-1 on regulatory T cells, by blocking antibodies, can lead to enhanced activation and expansion of immune-suppressive Treg within the tumor microenvironment [47]. Consequently, the ratio of PD-1^+^ Treg versus PD-1^+^ effector T cells within tumors might dictate the outcome of checkpoint antibody therapy directed at PD-1. A blockade of PD-1 may also enhance NK cell activity within tumors and modulate the activity of innate lymphoid cells (ILC), with some studies suggesting that NK cells help mediate the effects of anti-PD-1 blockade in metastatic melanoma and non-small-cell lung carcinoma [48,49,50]. The role of PD-1 on NK cells and ILCs in cSCC is largely unknown. Other preclinical studies using myeloid-specific deletion of PD-1 have shown a role for PD-1 on myeloid cells, in inhibiting anti-tumor immunity [51]. While PD-1 on tumor-infiltrating lymphocytes represents a clear therapeutic target, recent studies show that the blockade of PD-1 in tumor-draining lymph nodes contributes to the effectiveness of this checkpoint therapy [52]. A physiological role for PD-1 in the skin is suggested by the cutaneous adverse events associated with anti-PD-1 immunotherapy for metastatic cancers unrelated to the skin [53,54,55]. Immunotherapeutic targeting of PD-1 in advanced-stage melanoma or Merkel cell carcinoma has advanced to clinical trials, with promising response rates between 30 and 60% for monotherapy [56,57,58,59]. The response rates were higher in melanoma patients receiving both anti-PD-1 (nivolumab) and anti-CTLA-4 (ipilimumab) [59].

In contrast to melanoma, the number of clinical trials of anti-PD-1 therapy in cutaneous SCC have lagged behind, but are increasing rapidly (Table 1). The use of systemically administered anti-PD-1 (cemiplimab) therapy in a phase 2 clinical trial of primarily elderly patients with local advanced or metastatic cutaneous SCC has been reported [35,60]. It was observed that 47% of metastatic, cSCC patients and 44% of locally advanced, cSCC patients had an objective response to Cemiplimab treatment. Complete responses in both groups was relatively low (7–13%) and the rate of adverse events were consistent with other anti-PD-1 studies [61]. A long-term follow-up in this trial will be required, to accurately determine the duration of the response. These studies highlight the potential for targeting PD-1 in advanced cutaneous squamous cell carcinoma, while also suggesting room for therapeutic improvement and the need to characterize the PD-1 response in early SCC. One avenue to improved therapy would be the use of alternative checkpoint molecules as monotherapies, or in combination with anti-PD-1 therapy. The vast array of costimulatory (e.g., OX-40, ICOS) and coinhibitory checkpoints (e.g., TIM-3, TIGIT and LAG-3) being considered as targets for tumor immunotherapy have been reviewed elsewhere [29,45,62,63]. Next, we will consider a non-redundant co-inhibitory molecule, V-domain Ig suppressor of T-cell activation or VISTA, and a costimulatory protein, 4-1BB, both of which contribute to skin immunity.

## 5. V-Domain Ig Suppressor of T Cell Activation (VISTA or PD-1H)

VISTA is a novel immune checkpoint inhibitor of T cells and a member of the Ig superfamily [68]. VISTA sequencing data indicate that it shares sequence homology with PD-L1/2, and possesses a high level of structural homology between mouse and human, highlighting the possibility of a conserved signaling pathway and function [68,69]. VISTA is expressed across multiple immune cell types, including neutrophils, dendritic cells, macrophages, monocytes, NK cells, and T-cell subsets (both CD4 and, to a lesser extent, CD8 T cells), suggesting that its impact on T-cell function may involve both direct and indirect mechanisms [69,70]. T cells in VISTA-deficient mice are more prone to activation and show enhanced likelihood of induction of autoimmunity, suggesting that VISTA acts as a negative regulator of T-cell function. However, this may be an oversimplification, as VISTA has also been reported to co-stimulate immune responses in certain tissue contexts, suggesting that we need to understand more about the receptor–ligand interactions of VISTA [71]. Several ligands have been proposed for VISTA, including VISTA itself, VSIG3, Galectin-9 and PSGL-1, but further study will be required to determine the contribution of each molecule and the identity of other potential ligands [72,73,74,75]. Interestingly, VISTA-deficient mice also show a psoriasis-like inflammatory disease within the skin, which is consistent with a role for VISTA in skin immune responses. VISTA knockout mice have been reported to have increased antigen experienced T cells (CD44^hi^) in aged mice with skin inflammation (characterized by increased, localized, immune cell populations over time) [76,77]. Antagonist antibodies against VISTA also result in enhanced T-cell activation. While VISTA can be expressed on T cells, it is highly expressed on myeloid-derived cells, and can influence T cell function indirectly through assisting suppressive myeloid cell recruitment and limiting CD80 and IL-12/TNF-α production on antigen-presenting cells [78,79,80].

VISTA has been reported to have a role in regulating tumor progression [80]. Studies correlating increased VISTA expression with tumor progression have been noted in models of oral squamous cell carcinoma [81], cutaneous melanoma [82], and metastatic melanoma [83]. Wu et al. noted that poor prognosis in human oral squamous cell carcinoma was correlated with VISTA^hi^ CD8^low^ expression, in addition to elevated levels of myeloid-derived suppressor cells (CD11b and CD33^+^ cells) [81]. In melanoma, VISTA expression correlated with CD33 expression (a marker of myeloid-derived suppressor cells), and was associated with worse survival outcomes [84]. In this same study, co-expression of PD-1 and VISTA was associated with even worse survival rates. Yoon et al. demonstrated that death domain 1 alpha (later known as VISTA) is upregulated after p53 damage, to increase cell engulfment by tolerogenic phagocytes and suppress T-cell activity [85]. VISTA was also shown to be broadly expressed in cutaneous SCC tissue. This is most likely a means to clear the cellular environment of damaged cells in an effort to prevent autoimmune reactions. One could extrapolate a functional role for VISTA blockade in cancer from this study. By blocking VISTA, apoptotic tumor cells could persist in the environment, allowing for a source of antigens to be processed and presented by inflammatory phagocytes to T cells, enhancing the adaptive immune response.

Knowledge of VISTA function in cancer has been aided by the use of anti-VISTA antibodies (clones MIH63 and 13F3). Kondo et al. demonstrated that the MIH63 antibody in combination with the CTLA-4, but not PD-1, antibody marginally slowed tumor growth in a SCCVII transplantable tumor model (SCCVII: a poorly immunogenic and immunotherapy-resistant SCC model), relative to CTLA-4 or PD-1 monotherapy alone [86]. While MIH63 induced functional CD8^+^ T cells (CD8^+^/Eomes^+^/Ki67^+^), the presence of high numbers of Tregs may have dampened their ability to clear the tumor. A previous study by the same group, using the same tumor model, demonstrated that the depletion of Tregs using the anti-CD25 antibody completely cleared the tumor, indicating the importance of Tregs in modulating the tumor-killing response [87]. In a comprehensive study by Le Mercier et al., three melanoma models (transplantable B16OVA, B16-BL6 and the inducible PTEN/BRAF melanoma models) were treated with the anti-VISTA antibody 13F3 [80]. Monotherapy with 13F3 resulted in a significant delay in tumor growth across all three models, which is of interest, as the B16-BL6 model is known to be poorly immunogenic [80]. Other observations noted in the three models were as follows: increased tumor-infiltrating CD4 and CD8 cells, lower MDSC populations in TILs, and increased CD8^+^ IFN-γ^+^ cells compared to the Ig control [80]. A parallel series of experiments in the same study, using OTII^+^ CD4 T cells adoptively transferred into B16OVA tumor-bearing mice, indicated that the VISTA blockade decreased the percentage of induced, CD4^+^ FoxP3^+^ Treg cells amongst TILs, and in the draining lymph node. Finally, a study by Liu et al. determined that blocking antibodies against PD-L1 and VISTA can synergistically inhibit the growth of CT26 colon carcinoma cells injected subcutaneously in the flank [88]. This study also demonstrated that both PD-1 and VISTA single knockout mice exhibited chronic inflammation and spontaneous T-cell activation, suggesting a non-redundant role of these molecules in immune suppression.

Taken together, these observations suggest that VISTA has roles in modulating inflammatory responses, in addition to having a role in regulating anti-tumor immunity. Given that VISTA expression is associated with worse prognosis in skin cancers, and triggers non-redundant signaling pathways relative to PD-1, VISTA blockade represents a promising target for combination with PD-1 in cutaneous SCC. Both preclinical and clinical trials with this combination would be required to confirm any synergy and establish a safety profile.

## 6. 4-1BB (CD137) 

In contrast to the inhibitory signal of both PD-1 and VISTA, CD137 is acknowledged as a stimulatory signal for effector T cells. While CD137 is a cell surface molecule found on activated CD4^+^ and CD8^+^ T cells, a variety of immune cell subsets, including dendritic cells and macrophages, NK cells, NKT cells, eosinophils and mast cells, all express CD137 [89,90,91,92,93,94]. In dendritic cells, 4-1BB ligation induces DC maturation, improved antigen presentation and upregulation of T-cell signal 2 activation molecules (B7-1/2), in addition to the upregulation of several cytokines, including IL-6, IL-12 and IL-27 [89,95,96]. The role of 4-1BB on the other immune cell subsets is only beginning to be explored. The ligand for 4-1BB (4-1BBL or CD137L) is mostly expressed on B cells, macrophages and dendritic cells, consistent with a role in providing co-stimulation for T cells [90]. The binding of 4-1BB to 4-1BBL induces the NF-kB and MAPK signaling pathways, resulting in a variety of effects in T cells, including the upregulation of anti-apoptotic genes, production of pro-inflammatory cytokines (IFN-γ and IL-2), enhancement of CD8^+^ cytotoxicity (specifically perforin and granzyme), and enhancement of TCR signaling and memory cell formation [89,95]. In contrast, the engagement of 4-1BB on CD4^+^CD25^+^ Treg may lead to expansion of this suppressive subset [97].

In contrast to 4-1BB eliciting potent effector T-cell function, 4-1BB signaling has also been shown to reduce T-cell-mediated autoimmune responses in a variety of mouse models, including EAE, and Lupus, through activation induced death or anergy induction in autoreactive CD4 T cells. Specifically, in the EAE study, autoreactive CD4^+^ T cells were initially activated by antibody treatment, but then became more susceptible to activated-induced cell death as effector cells [98]. In the Lupus study, it was determined that anti-4-1BB treatment induced CD4^+^ T-cell anergy, and thus blocked T-cell-dependent humoral responses [99]. These studies, in addition to previous observations, indicate that agonist 4-1BB antibodies might elicit different responses from CD4 and CD8 T cells, and that the promotion of robust anti-tumor immunity might be a consequence of enhanced CD8 T-cell effector function.

While several studies have demonstrated the exciting potential of 4-1BB therapies across a variety of tumor models (breast, colon, ovarian, lung, liver, and melanoma models, reviewed in detail by Bartkowiak et al. [89]), there is concern for adverse health risks after intravenous administration. Increased levels of the liver enzymes aspartate aminotransferase (AST) and alanine aminotransferase (ALT), indicative of liver stress, were described in an anti-4-1BB antibody monotherapy study [100]. In a separate study evaluating combination immunotherapies in a B16F10 melanoma model, it was noted that anti-PD-1/4-1BB combination efficiently induced a synergistic anti-tumor response, evidenced by robust CD8^+^/Treg ratios and the upregulation of anti-tumor response genes (CD3ε, CD8α, IFN-γ, Eomes), but with toxicity related to the anti-4-1BB antibody alone [101]. A study by Kocak et al., using anti-CTLA-4/4-1BB combination therapies, also induced CD8^+^ T-cell-mediated tumor regression in a subcutaneous MC38 colon cancer model [102]. This study also indicated that 4-1BB monotherapy resulted in liver inflammation, which was surprisingly reduced by using a combination of antibodies directed at both 4-1BB and CTLA-4. The antibody combination increased Treg function, which may have decreased the liver inflammation, while still allowing for tumor regression at a different site. The degree of toxicity with various anti-4-1BB antibodies is associated with the strength of the agonist activity and the isotype of the antibody [103]. Engineering anti-4-1BB antibodies to minimize liver toxicity, by targeting the tumor microenvironment, is currently being investigated [104].

Within skin cancers, the exploration of anti-4-1BB antibody therapy has mostly been directed against melanoma [105]. MART-1-specific CD8 T cells were shown to have upregulated 4-1BB on the cell surface, and in vitro effector function was associated with 4-1BB-expressing cells [106]. Clonally expanded, tumor-specific T cells were also found amongst CD137^+^ CD8^+^ tumor-infiltrating lymphocytes in a B16F10 melanoma model [107]. Clinical trial progress in advanced melanoma has been hindered by adverse events, although low doses of urelumab (<1 mg/kg) seem to be tolerated [108]. A second antibody targeting CD137, utomilumab, was also found to be safe, and induced an objective response (2/15) in a limited number of Merkel cell carcinoma patients [109]. In contrast, the use of anti-CD137 antibodies, either alone or in combination, in cSCC is limited.

Cumulatively, antibodies against 4-1BB, either alone or in combination, show great preclinical promise in activating anti-tumour T cells in a variety of cancers. However, given that the systemic administration of 4-1BB carries a risk of liver toxicity, delivery strategies need to be optimized. The skin is an easily accessible site, allowing for localized treatment of skin cancer in the future, with reduced risk of systemic adverse events.

## 7. Conclusions

**Figure 1:**
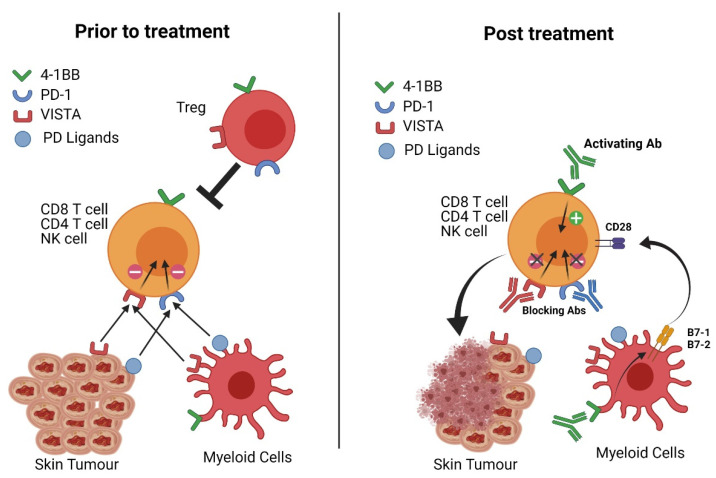
A model of the key interactions that are proposed to enhance the activity of T cells/NK cells within the tumor microenvironment after treatment with antibodies targeting PD-1, VISTA and 4-1BB. Prior to antibody treatment (left side), CD4/CD8 T cells and NK cells receive negative signals from interactions with ligands on the cancer and myeloid cells. In addition, there is a proposed lack of positive signals through 4-1BB and the presence of Treg cells that inhibit T cell/NK cell activity. Following antibody treatment (right side), T cells/NK cells are activated due to the antibody blocking of PD-1 and VISTA and the agonistic activity of 4-1BB. In addition, anti-4-1BB antibody is known to delete Treg cells (no longer shown in the diagram) and induce costimulation (such as B7 family members) on dendritic cells allowing activating signals through CD28. The end result is destruction of the tumor by T/NK cells. Note that not all T/NK cells will express all three receptors simultaneously leading to different levels of functional restoration within individual T/NK cells in the tumor microenvironment (image created with BioRender.com (accessed on 12 May 2021)).

There is no doubt that checkpoint inhibitor therapy has revolutionized our treatment options for a variety of tumors. While some patients undergoing these treatments can show regression of large tumors, others fail to fully respond. This has led to a rapid expansion of combination treatments where checkpoint inhibitors are combined with more conventional therapies, such as chemotherapy or radiotherapy. These combinations ensure that the tumor is attacked from multiple angles, thus increasing the chance of curative therapy. Similarly, when considering combinations of checkpoint therapy, multiple targets in the tumor microenvironment should be considered. Combinations of antibodies against PD-1, 4-1BB and VISTA fulfil this brief by not only targeting positive and negative signaling pathways on CD8 cytotoxic T cells, but also impacting on CD4 effector T cells, NK cells, regulatory T cells and myeloid-derived suppressor cells within skin cancers (Figure 1). While this approach is useful in tumors such as skin cancer, where inflammatory infiltrate and high mutational burden is frequently observed (“hot” tumours), combination checkpoint therapy will likely be more challenging in tumor microenvironments that are devoid of immune cells. In these tumors, the additional use of immune chemoattractants or the induction of immunogenic tumor cell death will be important.

**Figure 2:**
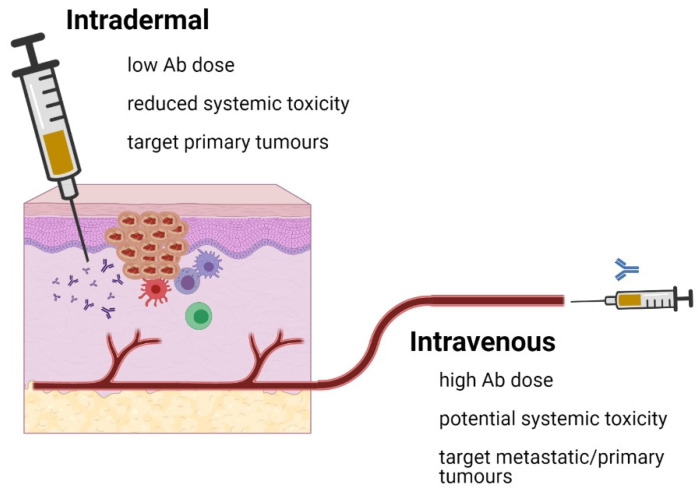
Local injection of checkpoint antibodies in skin cancer could enhance efficacy and safety of these treatment protocols. The diagram summarizes the key features of local vs. systemic application of checkpoint antibodies (image created with BioRender.com (accessed on 12 May 2021)).

The successful application of combination checkpoint therapy to skin cancers requires careful consideration of systemic toxicities often seen as autoimmune responses. This has been an issue with some anti-4-1BB antibodies as a single intravenous agent, but may be exacerbated by the use of multiple checkpoint antibodies in combination. Skin cancer patients with pre-existing autoimmune disease may be particularly susceptible. Conversely, solid organ transplant patients on high-dose, immunosuppressive drugs with cutaneous SCC may not benefit from checkpoint antibodies designed to reactivate tumor-specific T cells. In localized, primary cSCC the accessibility of the tumor requires a re-evaluation of the need for intravenously delivered therapy at high doses. While this traditional approach is clearly useful in the setting of metastatic skin cancer (antibody is distributed widely throughout the body), it may not be required for primary skin tumors where reactivation of infiltrated T cells is required. Given that some checkpoints mainly act at the level of T-cell priming in the lymph node (e.g., CTLA-4), while others alter the behavior of activated T cells in the tissue (e.g., PD-1), it will be important to match the checkpoint antibody with the required location. However, focusing on checkpoint antibodies that alter the behavior of activated T cells (such as antibodies directed against PD-1/4-1BB/VISTA), there is the potential for local treatment of the tumor, with much lower doses of antibody to minimize the systemic toxicities while preserving the anti-tumor efficacy (Figure 2). This does not exclude the possibility of small amounts of intradermal antibody draining to the lymph nodes and promoting a broader systemic T-cell response. Currently, there are a limited number of clinical trials examining this approach for skin cancer (clinicaltrials.gov (accessed on 19 April 2021), trial #NCT03889912). Future research should examine antibody dosing, toxicity and potential abscopal effects generated by the local treatment of individual cSCCs. Local injection of combination antibody could also be used as a neo-adjuvant therapy with surgery, to help reduce tumor size or improve accessibility prior to surgical removal [110]. Adjuvant effects may also be seen if the checkpoint antibody enhances local dendritic cell maturation en route to the local lymph nodes. Given that injected antibodies have a set half-life, these adjuvant effects might be important in promoting the continued generation of new anti-tumor T-cell responses in the absence of an antibody. Alternatively, if antibodies against PD-1, 4-1BB and VISTA promoted long-term, T-cell memory, then the need for reapplication of therapy would be diminished. The development of memory T cells after checkpoint therapy in cSSC warrants further investigation.

In conclusion, PD-1, 4-1BB and VISTA provide useful immunotherapeutic targets in skin cancer, and their combined use, applied locally or systemically, could provide improved treatment options for cutaneous SCC into the future.

## Figures and Tables

**Table 1:** Clinical trials ^1^ for antibodies directed against PD-1/PD-L1, CD137, VISTA in cutaneous SCC.

Therapy Target	Therapy	Indication	Clinical Trial #	Clinical Trial Status	Reference
PD-1	Pembrolizumab	Multiple, including SCC patients with poor prognosis and progression on standardized therapies	NCT02721732	Phase II	[64]
PD-1	Pembrolizumab	Recurrent and/or metastatic cSCC	NCT03284424	Phase II	[65]
PD-1	Pembrolizumab	Recurrent cSCC in patients not curable by surgery or radiation	NCT02964559	Phase II	[66]
PD-1	Pembrolizumab Radiation	Postoperative radiotherapy for cSCC of head and neck	NCT03057613	Phase II/completed	
PD-1	Pembrolizumab	Unresectable/metastatic squamous cell carcinoma	NCT02883556	Phase II	[67]
PD-1 EGFR	Pembrolizumab in combination with Cetuximab	cSCC of head and neck	NCT03082534	Phase II/recruiting	
PD-1 C5a	Pembrolizumab in combination with IFX-1(anti-C5a Ab)	Locally advanced or metastatic cSCC	NCT04812535	Phase II/not yet recruiting	
PD-1	Pembrolizumab	High-risk, locally advanced cSCC following surgery and radiation	NCT03833167	Phase III/recruiting	
PD-1	Pembrolizumab	PD-1 naïve cSCC	NCT04808999	Phase II/recruiting	
PD-1	Cemiplimab	Locally advanced or metastatic cSCC	NCT02383212	Phase I/ metastatic or locally advanced SCC phase II metastatic cSCC	[35]
PD-1	Cemiplimab	Locally advanced or metastatic cSCC	NCT02760498	Phase II/recruiting	[60]
PD-1	Cemiplimab	Recurrent cSCC	NCT03889912	Phase I/active/not recruiting	
PD-1	Cemiplimab	cSCC stage II to IV	NCT04154943	Phase II/recruiting	
PD-1	Cemiplimab	Immunocompromised patients with unresectable locally recurrent and/or metastatic cSCC	NCT04242173	Phase II recruiting	
PD-1	Cemiplimab in conjunction with RP-1 (modified HSV-1)	Locally advanced/metastatic cSCC. Combination with RP-1 intratumourally	NCT04050436	Phase II/recruiting	
PD-1	Cemiplimab	Recurrent stage III-IV cSCC of head and Neck	NCT03565783	Phase II/recruiting	
PD-1 SAR444245	Cemiplimab in conjunction with SAR444245 (rhIL-2)	Locally advanced or metastatic cSSC	NCT04913220	Phase I/II/not yet recruiting	
PD-1	Cemiplimab	High-risk, stage III cSCC	NCT04632433	Phase II/recruiting	
PD-1	Cemiplimab	High-risk cSCC before and after surgery	NCT04428671	Phase I/recruiting	
PD-1	Cemiplimab	High-risk cSCC after radiation and surgery	NCT03969004	Phase III/recruiting	
PD-1 TLR-9 agonist	Cemiplimab or Pembrolizumab with Cavrotolimod (TLR-9 agonist)	Advanced or metastatic cSCC. Combination with cavrotolimod intratumourally	NCT03684785	Phase I/II/recruiting	
PD-1	Cemiplimab In conjunction with Everolimus/Sirolimus/Prednisone	Advanced cSCC in participants who have previously received an allogeneic hematopoietic stem cell transplant or kidney transplant	NCT04339062	Recruiting	
PD-1	Cemiplimab	High-risk localized/locally recurrent/resectable cutaneous cSCC	NCT04315701	Recruiting	
PD-1 EGFR	Cemiplimab Pembrolizumab ASP-1929 (Anti-EGFR Photoimmunotherap)	Recurrent or metastatic head and neck SCC or advanced or metastatic cSCC in EGFR-expressing tumours	NCT04305795	Recruiting	
PD-1	Nivolumab	Advanced cSCC	NCT03834233	Active/not recruiting	
PD-1 CTLA-4	Nivolumab+/− Ipilimumab	Resectable stage III-IVa cSCC	NCT04620200	Phase II/recruiting	
PD-1	Nivolumab	Locally advanced/metatstatic cSCC	NCT04204837	Phase II/not recruiting	
PD-L1 EGFR	Avelumab+/− Cetuximab	Advanced cSCC	NCT03944941	Phase II/recruiting	
PD-L1	Avelumab in combination with radiation	Unresectable cSCC	NCT03737721	Phase II/recruiting	
PD-L1	Atezolimumab Cobimetnib (MEK Inhibitor)	cSCC	NCT03108131	Active, not Recruiting	
PD-L1	Atezolimumab NT-I7(rhIL-7-Fc)	cSCC	NCT03108131	Recruiting	
PD-L1	Atezolimumab	cSCC	NCT04710498	Not yet recruiting	
VISTA	CI-8993	Advanced solid tumour malignancies	NCT04475523	Recruiting	

^1.^ As reported at clinicaltrials.gov (accessed on 19 April 2021). Some studies where evaluation of cSCC was not a primary outcome were omitted.
